# Koch’s curse: How models of extreme pathology bias studies of host–pathogen interactions

**DOI:** 10.1371/journal.ppat.1011997

**Published:** 2024-03-15

**Authors:** Kalyan K. Dewan, Eric T. Harvill

**Affiliations:** Department of Infectious Diseases, College of Veterinary Medicine, University of Georgia, Athens, Georgia, United States of America; University of Massachusetts, Worcester, UNITED STATES

## Introduction

The 19th century was a turbulent period in the emerging field of microbiology. The startling claims of the existence of “animalcules” by Antoine von Leeuwenhoek in 1676 were being validated through advances in sterile procedures and techniques for isolating microbes to purity. Louis Pasteur presented his germ theory of disease transmission (1859). Robert Koch observed the anthrax bacillus in the blood of diseased cows could cause disease in mice [1]. But, there was not yet consensus among disparate approaches to definitively relate a pathogen to the specific disease it caused. It was in this chaotic period that Robert Koch (1890) introduced a compelling approach to systematically determine the etiological agents of infectious diseases, now enshrined in the illustrious Koch’s postulates: (i) The pathogen (infectious agent) must be found in diseased but not healthy individuals; (ii) The pathogen should be isolated from a diseased host; (iii) A healthy host inoculated with the cultured pathogen must reproduce the disease; and (iv) The pathogen must be reisolated from the inoculated, diseased host and be identical to the original pathogen. These guidelines introduced rigorous and objective criteria to causally link a culturable microorganism to a specific disease.

Studying diseased animals as a proxy for human diseases dates back to antiquity and was widely employed in the 19th century. But satisfying Koch’s postulates presented 2 substantial challenges: (1) Most human pathogens, when inoculated in small numbers, do not colonize and grow effectively enough to induce similar pathology in animals; and (2) Symptoms of low-level infections are generally minor and more variable, making them difficult to accurately replicate in animals. These combined problems were conveniently solved by the delivery of large doses of pathogens that resulted in extreme forms of disease. Microbes delivered in large doses were evaluated for their “pathogenesis” and “virulence” by relatively simple and consistent measures of extreme disease. The approach was procedurally sound and results highly reproducible and soon spread across the scientific world. By the 1950s, an entire field of infectious disease research called Bacterial Pathogenesis, building directly on Koch’s postulate-based approaches, routinely used lethal or near-lethal inoculation doses to induce severe pathology in animals, providing much of what we know about pathogens and the roles of their specific “virulence factors” in extreme forms of disease.

As our understanding has grown, along with new technologies and approaches, it is becoming more evident that there are limitations to a single-minded focus on extreme disease. There are now gathering reports that critical aspects of host–pathogen interactions are masked by very high inoculation doses. For example, comparing *Mycobacterium tuberculosis* infections following traditional high-dose aerosol exposure to lower doses that more closely model the pathology of human tuberculosis [2] results in different effects on the hosts [3]. Similarly, in a human challenge study, varying viral inoculum doses from 4,800 to 0.48 reverse transcription units altered symptom severity and transmission dynamics of norovirus infections [4]. Similar dose-dependent issues have been reported in COVID-19 challenge studies in monkeys [5], supporting the view that a careful consideration of the impact of challenge dose may improve our understanding of pathogen–host interactions [6].

## Pathogens modulate disease to optimize success

For most successful pathogens, there are both benefits and risks of inducing severe pathology that must be delicately balanced. While some symptoms of disease can contribute to transmission (i.e., diarrhea, rhinorrhea, or coughing), extreme pathology can result in morbidity and mortality that reduce the chance for transmission, which at the population level can result in an evolutionary dead end for the pathogen. While extremely virulent pathogens do arise in humans, often spilling over from other animal populations and causing short-term outbreaks or plagues, it is important to consider the evolutionary trade-offs that tend to modulate virulence over time [7]. To be successful, these organisms must do much more than simply cause extreme disease symptoms.

Successful commensals and pathogens often have evolved highly specialized mechanisms that mediate complex interactions with the host to establish infection. These can mediate their reaching the appropriate host organ (e.g., inhalation, ingestion), attaching to the biological substratum, invading and accessing host nutrients to divide and spread within, and ultimately among hosts. Microbes carefully choreograph the deployment of factors that disrupt or modulate host defenses without necessarily inducing overt disease symptoms. Generating and optimizing effective propagative means (aerosolized droplets, fomites, etc.) and the colonization by the smallest effective propagule (idealized as a single organism) can increase transmissibility/contagiousness to the point where inducing extreme symptoms in the host is not necessary for success. Importantly, these and other critical aspects of host interactions may be initiated by the first few colonizing pathogens reaching a target organ. Potentially subtle but critical early pathogen–host interactions are likely to be substantially different when an overwhelming number of the microbe are delivered in a high-dose bolus directly to sensitive organs to induce severe disease, as in conventional Bacterial Pathogenesis studies.

## Pertussis as a case in point

Pertussis came to prominence in relatively recent recorded history; descriptions of an epidemic with symptoms matching pertussis appeared in the 15th century [8]. The first description of pertussis as an independent disease (“*sui generis*”) is believed to be in the 17th century [9], but by the 19th century, pertussis was widely recognized as extremely virulent in infants and, along with measles, dreaded as a major cause of infant mortality [10]. The gram-negative coccobacilli, *Bordetella pertussis*, was first identified by Jules Bordet and Octave Gengou in 1906 [11], at a time when the pathogen was strongly suspected, but not yet proven to be the causative agent of pertussis. In 1912, Mallory and Hornor satisfied Koch’s postulates using dogs to ascribe *B*. *pertussis* as the causative agent of pertussis [12].

Classical pertussis is described as an acute infection of the upper respiratory tract, though its symptoms can persist several months. The initial catarrhal stage, recognized early to be a highly contagious period, is deceptively mild, with occasional coughing and sneezing for about 2 weeks. Coughing gradually intensifies over weeks and takes a violent turn to the paroxysmal stage characterized by fits of spasmodic coughing, often accompanied with vomiting and cyanosis in younger patients. But the organism generally cannot be cultured by the time severe symptoms arise, raising questions about whether the most severe forms of these symptoms are critical to the biology of the organism. Furthermore, both primary symptoms and much of the fever and inflammation derive from secondary infectious complications. Despite these caveats, the Koch’s postulates-based approach, overwhelmingly focused on simulating severe, lower respiratory pathology in animals within days of a very large inoculum, has produced critical advances, the most important being vaccines that prevent severe disease.

### Vaccines against pertussis

The important goal of developing effective vaccines to prevent the most severe disease required effective models of those. Thus, the extraordinary high-dose inoculum, roughly a million colony-forming unit (CFU) delivered deep into the lungs in aerosol or liquid, was increasingly viewed as the standard for all such studies. This very high-dose challenge largely overrides host specificity to induce a rapid major inflammatory response in animal models and is marked by high bacterial loads deep in the lungs with leukocytosis and pulmonary hypertension, recapitulating the most severe form of disease in infants and satisfying Koch’s postulates. These approaches, particularly using mice [13], also powered the development and introduction of very effective whole-cell pertussis (wP) vaccines that dramatically brought under control what had been one of the great scourges of the 20th century, among the great success stories in the vaccine control of infectious diseases. As the perils of pertussis faded from public memory, the occasional fever and swelling the wP vaccines induced came under greater scrutiny and, fueled by false reports of neurological side effects [14], led to widespread resistance against the vaccine in several countries [15]. Rather than revert to the horrors of the prevaccine era, safer and less reactogenic acellular pertussis (aP) vaccines composed of purified proteins as antigens were then developed using the then widely accepted mouse models of high-dose challenge focusing on severe pathology in the lung [16]. The new vaccines protected against severe disease and induced significantly reduced side effects in humans and are now the only versions of pertussis vaccines delivered in most developed countries, including the United States.

Though aP vaccines reduced side effects and increased compliance, pertussis cases have gradually risen since their rollout. Limited nonhuman primate studies confirm they prevent the most severe pneumonic forms of pertussis but also suggest a more subtle and insidious problem; aP vaccines are much less effective in preventing the colonization, growth, and asymptomatic persistence of *B*. *pertussis* in the upper respiratory tract, allowing colonization, ongoing shedding, and continued transmission to new hosts [17]. The models used to develop and test aP vaccines, following the high-dose/severe pulmonary disease approach of Koch’s postulates, effectively simulated severe disease, but did not simulate the course of the much more common less severe forms of infections, and so did not detect this shortcoming. Exacerbating the emerging problem, aP vaccines appear to suppress the distinct symptoms that allow effective diagnosis of pertussis, reducing the opportunity for quarantine and treatment, and further contributing to increased transmission [18].

## Understanding pertussis infection

It is pertinent to note that by the time the classical coughing symptoms of pertussis emerge in adults, the sensitivity of detecting the pathogen by culture is very low, suggesting that *B*. *pertussis* has completed its “life cycle”, having already shed and transmitted efficiently to unsuspecting hosts. Importantly, these transmission events that seed new *B*. *pertussis* infections likely involve smaller numbers of bacteria that colonize and grow while neutralizing the formidable host mucosal immune response. But these important aspects of the infectious process are not well replicated in the high dose, extreme virulence of the conventional model. Establishing animal infection models using lower bacterial numbers to study these aspects of natural infection has been challenging for a list of reasons. But we reasoned and argued that a broadly usable low-dose infection model would enable assays to measure aspects of infection that cannot be accurately observed when measured in the conventional high dose, extreme virulence model.

**Fig 1:**
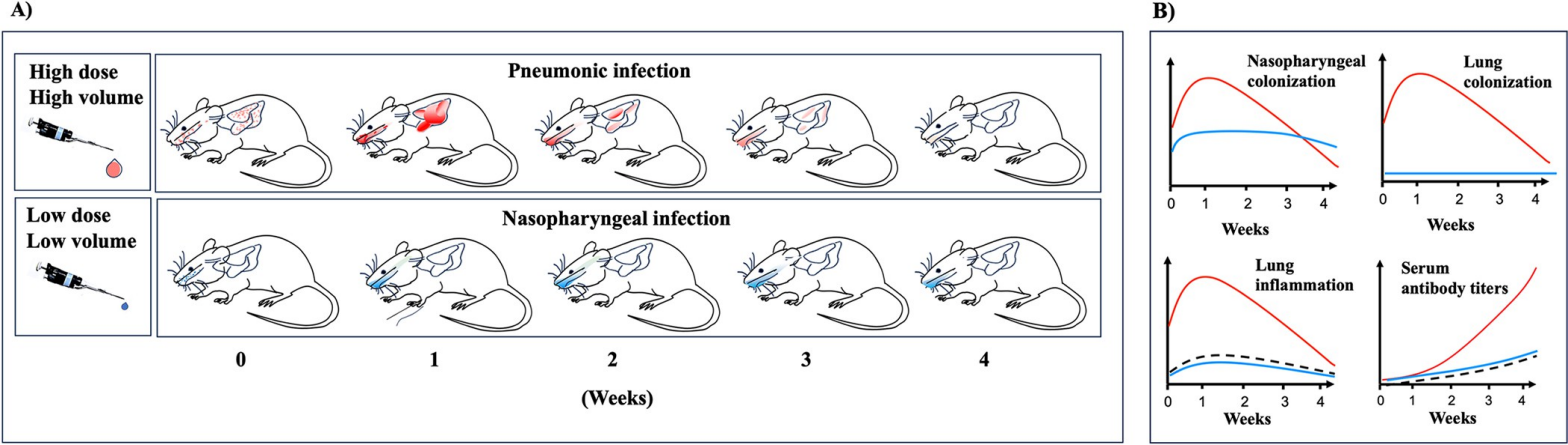
(**A**) Schematic highlighting differences in growth, spread, and persistence of *B*. *pertussis* in regions of the respiratory tract differ based on inoculation dose. (**B**) Graphical representation of colonization and host response based on high- and low-dose inoculations (red, high dose high volume; blue, low dose low volume). Black dashed lines indicate an approximation of the adult human response during typical pertussis. (Figure is a compilation of images sourced from open software source https://commons.wikimedia.org).

Several discoveries have recently made more natural low-dose infection models more feasible. We discovered that perturbing the nasal microbiota of mice with small doses of topical antibiotic allowed even a few hundred CFUs of *B*. *pertussis* to efficiently colonize the nasal cavities of mice [19]. When delivered at these low doses, *B*. *pertussis* remained predominantly localized within the nasopharynx, consistent with the description of *B*. *pertussis* primarily being an upper respiratory tract pathogen [20]. Further, after growing to tens of thousands in numbers over the course of the first week, they persisted at moderate numbers over at least 4-week studies, inducing a relatively modest immune response. Importantly, these findings highlight the ability of *B*. *pertussis* to modulate the host immune response during this initial expansion, hallmarks of the predominantly mild pertussis cases that predominate in healthy adults. These observations are in stark contrast to high-dose pneumonic models in naïve mice, in which severe acute pulmonary pathology induces extremely robust adaptive immune responses, with antibody titers often in the tens of thousands (Fig 1). Interestingly, persistence with no overt symptoms appears to be a common outcome of many subclinical pertussis infections, in which the serological criteria for prior infection require less than 10-fold increase in antibody titer. The low-dose inoculation approach better simulates these subclinical infections and also allows for the accurate measurement of the sometimes-subtle contributions of individual bacterial factors to aspects of the infectious process that are obviated by the high-dose approach. Delivering bacteria in large numbers deep in the lungs is likely to bypass the need for bacterial factors involved in initial attachment to upper respiratory epithelia, early gradual growth while modulating the local innate/inflammatory response, spread within and between respiratory organs, and modulating adaptive immunity to persist for several weeks. Conditions that better replicate the more gradual and less severe course of most infections may be better suited to observe and measure the contributions of bacterial factors involved, and the more subdued host response to the natural infection process.

## Conclusions

As we look more deeply and holistically at host–pathogen interactions, the characteristics of existing animal models can be increasingly refined to better simulate the specific aspects of their biology we hope to study and understand. Such studies can be guided in part by an appreciation of the evolutionary pressures that shape most pathogens toward inflicting less severe disease and/or toward commensalism, to allow persistence in a stable host population over evolutionary time. While controlling/eliminating extreme human disease remains a primary goal, the singular focus on the study of only extreme disease is likely to overlook other important aspects of pathogen–host interactions, as illustrated by the story of pertussis. A broader view, inclusive of more moderate pathogen challenges, and perhaps other approaches to simulate natural infections, may induce less measurable pathology, but may reveal other aspects of the pathogen biology that are critical to its success, and thereby present additional effective targets and approaches to preventing infectious diseases. There is substantial need for alternative animal models that focus on how pathogens successfully colonize initially in small numbers, grow gradually, modulate immunity to persist, be shed, and transmit among hosts, with or without inducing symptomatic pathology. We submit that the prescient genius of Koch, who was among the first to point out problems and limitations of his postulates, would agree that more moderate challenges that better simulate natural infection are likely to be highly complementary to conventional models focused on simulating extreme disease.
